# Beyond morphology: functional imaging of the liver with gadoxetic acid-enhanced magnetic resonance imaging

**DOI:** 10.1007/s00261-026-05623-w

**Published:** 2026-06-22

**Authors:** Eleonora Salles-Silva, Sthefane Louise Gomes Nunes, Rosana Souza Rodrigues, Antonio Luis Eiras de Araújo, Renata Mello Perez, Giuseppe D’Ippolito, Caroline Lorenzoni Almeida Ghezzi, José Hugo Mendes Luz, Viviane Brandão Amorim, Daniella Braz Parente

**Affiliations:** 1 Radiology Department, Fleury S.A. (Brazil), Rio de Janeiro, Brazil; 2 Radiology Department, Federal University of São Paulo, São Paulo, Brazil; 3 Radiology Department, D’Or Institute for Research and Education, Rio de Janeiro, Brazil; 4 Radiology Department, Federal University of Rio de Janeiro, Rio de Janeiro, Brazil; 5 Hepatology Department, Federal University of Rio de Janeiro, Rio de Janeiro, Brazil; 6 Hepatology Department, D’Or Institute for Research and Education, Rio de Janeiro, Brazil; 7 Radiology Department, Fleury S.A. (Brazil), Rio de Janeiro, Brazil; 8 Radiology Department, Hospital de Clínicas de Porto Alegre, Porto Alegre, Brazil; 9 Radiology Department, Hospital Moinhos de Vento, Porto Alegre, Brazil; 10 Interventional Radiology Department, Instituto Nacional do Câncer, Rio de Janeiro, Brazil; 11 Radiology Department, Instituto Nacional do Câncer, Rio de Janeiro, Brazil

**Keywords:** Gadoxetic-acid; Magnetic resonance imaging; Functional Liver Imaging Score; Liver function; Chronic liver disease.

## Abstract

Chronic liver diseases (CLD) are highly prevalent. Early diagnosis and staging of hepatic dysfunction are essential for optimal treatment decisions. While conventional imaging primarily evaluates morphology and focal liver lesions, gadoxetic acid (GA)-enhanced magnetic resonance imaging (MRI) provides additional functional information by assessing hepatocellular uptake and biliary excretion. Hepatocyte uptake depends largely on sinusoidal membrane transporters (OATP1B1/1B3), while excretion into bile canaliculi is mediated by MRP2. In liver dysfunction and cirrhosis, both processes may be impaired, resulting in measurable changes on hepatobiliary phase (HBP). This review summarizes the biological rationale, MRI protocol, and quantitative indices, including RLE, REI, HUI, rHUI, SIR, CUI, LSI, as well as the qualitative Functional Liver Imaging Score (FLIS). It also highlights key clinical applications, including prognostication in advanced CLD, preoperative risk stratification for post-hepatectomy liver failure, evaluation in the setting of embolization and locoregional therapies, and transplant assessment, along with emerging roles in metabolic dysfunction-associated steatotic liver disease. Finally, future directions are discussed, including T1 mapping and AI-based whole-liver quantification.

## Introduction

Chronic liver disease (CLD) encompasses a broad spectrum of etiologies and degrees of hepatic impairment, often requiring longitudinal monitoring, accurate staging, and precise functional characterization. Conventional assessment relies on clinical and laboratory scores, such as Child-Pugh (CP), Model for End-Stage Liver Disease (MELD), and Albumin-Bilirubin (ALBI) scores, and ultrasound, computed tomography, MRI, elastography, biopsy, and indocyanine green (ICG) clearance test. Although these tools provide essential structural and biochemical information, many critical decisions in hepatology, oncology, liver surgery, and interventional radiology depend not only on morphologic findings but also on the global and regional functional reserve of the liver.

Hepatospecific contrast-enhanced MRI enables hepatic imaging to move “beyond morphology” by incorporating hepatocyte-specific contrast kinetics into routine examinations. Through its dual extracellular and hepatobiliary properties, hepatobiliary phase allows simultaneous evaluation of vascular dynamics, hepatocellular uptake, and biliary excretion within a single comprehensive study. This functional capability provides a noninvasive assessment of liver function through quantitative and qualitative approaches.

## Biologic rationale and pharmacokinetics of hepatospecific contrast

Gadoxetic acid (Gd-EOB-DTPA) is a gadolinium chelate containing a lipophilic ethoxybenzyl moiety that confers both extracellular and hepatocyte-specific properties [1]. Because of its higher protein binding and T1 relaxivity compared with conventional extracellular agents, it is administered at a lower approved dose of 0.025 mmol/kg versus 0.1 mmol/kg for nonspecific gadolinium agents [2].

A key pharmacokinetic feature is dual elimination, with approximately 50% of the administered dose taken up by hepatocytes and excreted into bile and the remainder cleared by renal glomerular filtration [2, 3]. In mild to moderate hepatic dysfunction, renal clearance increases to compensate, whereas hepatic elimination may increase in the setting of renal impairment [2]. After intravenous injection, the agent initially distributes within the intravascular and interstitial compartments during the arterial and portal venous phases, similar to extracellular chelates [3]. Active hepatocellular uptake begins during the transitional phase and results in peak hepatobiliary enhancement at approximately 20 min, which may persist for up to 2 h [1, 3].

Hepatocellular handling of Gd-EOB-DTPA depends on membrane transporters. Uptake across the sinusoidal membrane is mediated by OATP1B1 and OATP1B3 [2, 3], whereas biliary excretion occurs via the ATP-dependent transporter MRP2, also termed cMOAT [2, 4]. Sinusoidal retrograde flux may occur due to the bidirectional nature of OATP transport, with additional efflux mediated by basolateral transporters such as MRP3 and MRP4, particularly in pathologic states [2, 3].

Hepatobiliary phase (HBP) enhancement reflects the dynamic interplay between hepatocellular uptake, biliary excretion, and overall functional hepatocyte mass [3]. In patients with preserved liver function, biliary excretion can be detected within minutes, and a 20-minute delay is sufficient for biliary assessment [1, 4].

In cases of fibrosis and cirrhosis, there is a decrease in the number of working hepatocytes and an ongoing process of fibrotic tissue remodeling, which leads to less parenchymal enhancement during the HBP. This reduction may also be affected by changes in the expression and function of hepatocellular transporters. Studies using experimental rat models of cirrhosis show similar findings [3, 5]. A liver with steatosis and steatohepatitis also shows downregulation of sinusoidal uptake transporters [6].

In addition to these molecular mechanisms, impaired hepatic elimination contributes to prolonged vascular enhancement due to delayed systemic clearance [4]. Taken together, these data reinforce the value of GA-enhanced MRI as a noninvasive imaging biomarker that combines morphological with functional assessment of the hepatobiliary system [3].

Gadobenate dimeglumine is another gadolinium-based contrast agent with partial hepatobiliary excretion that may provide functional information about hepatocyte uptake and biliary excretion. However, compared with gadoxetic acid, its hepatobiliary uptake is substantially lower, with only approximately 3–5% of the injected dose excreted through the biliary system. As a result, the hepatobiliary phase is typically acquired later, usually around 60–120 min after injection [7, 8]. While gadobenate dimeglumine may provide complementary information on hepatobiliary function, its weaker hepatocellular uptake, delayed imaging requirement, and less extensive validation limit its role as a dedicated functional liver imaging agent. Although both agents can demonstrate hepatobiliary enhancement, gadoxetic acid provides stronger and earlier hepatocyte-specific enhancement, therefore it is the preferred contrast agent for routine and research applications focused on liver function assessment.

## MRI acquisition

Liver MRI for the assessment of hepatic function can be performed on 1.5T or 3 T systems using phased-array body or abdominal coils. GA is administered intravenously at a standard dose of 0.025 mmol/kg (0.1 mL/kg) at a rate of 1.0–2.0 mL/s, followed by a 20–30 mL saline flush. The imaging protocol includes pre-contrast sequences and multiphasic dynamic T1-weighted fat-suppressed three-dimensional gradient-echo acquisitions obtained during the arterial, portal venous, and transitional phases, followed by a 20-minute HBP. Additional sequences typically include axial in- and opposed-phase T1-weighted imaging, T2-weighted fast spin-echo imaging with magnetic resonance cholangiopancreatography (MRCP) when indicated, and diffusion-weighted imaging (DWI) with multiple b values (0–1000 s/mm²) with corresponding apparent diffusion coefficient (ADC) maps. A summary of the technical parameters, contrast administration, and suggested phase timing across studies is provided in Table 1 [3, 6, 9–17].

**Table 1:** Comparative Table: Gadoxetic Acid MRI Protocols (1.5T vs. 3 T)

Parameter	1.5T Protocol	3 T Protocol
Coil	8–12 channel phased-array body coil	6-element abdominal + spine coil; 12–32 channel body coil
Contrast dose	0.025 mmol/kg (0.1 mL/kg)	0.025 mmol/kg (0.1 mL/kg)
Injection rate	1.0–2.0 mL/s	1.0–2.0 mL/s
Saline flush	20-30mL	20-30mL
Dynamic phases	Precontrast; Arterial: 25–30 s (bolus-tracking or multi-arterial if possible); Portal venous: 60–70 s Transitional (equilibrium): 120–180 s; Hepatobiliary phase: 20 min	Precontrast; Arterial: 25–30 s (bolus-tracking or multi-arterial if possible); Portal venous: 60–70 s Transitional (equilibrium): 120–180 s; Hepatobiliary phase: 20 min
T13D Gradient-echo	In- and Opposed-Phase Imaging	In- and Opposed-Phase Imaging
T2 imaging	Fast spin-echo	Fast spin-echo
DWI/ADC map	b values typically 0, 50, 300–600, and/or 1000 s/mm²; slice 4–6 mm	b values typically 0, 50, 300–600, and/or 1000 s/mm²; slice 4–6 mm
Additional techniques	MRCP; PDFF; MRE	MRCP; PDFF; MRE

DWI = diffusion-weighted imaging; ADC = apparent diffusion coefficient; MRCP = magnetic resonance cholangiopancreatography; PDFF = proton density fat fraction; MRE = magnetic resonance elastography

## Quantitative assessment of liver function on GA-enhanced MRI

There are quantitative and semiquantitative methods for assessing liver function based on analysis of the 20-minute HBP of GA-enhanced MRI, as well as complex mathematical models such as time to peak (Tpeak), elimination half-life (t½), and liver and vascular input concentration-time curves. However, these approaches require prolonged acquisition times, are susceptible to respiratory motion artifacts, and lack standardization across MR platforms. As a result, it is still challenging to determine standard reference values for normal and impaired liver function, so these values are mainly used in research settings at present [3].

Therefore, simplified approaches have been proposed to estimate liver function based on signal intensity (SI) behavior during the 20-minute HBP, and these methods have been validated in multiple studies. These include relative liver enhancement (RLE), hepatic uptake index (HUI), contrast uptake index (CUI), and the liver-to-spleen index (LSI) [3].

Below, every method will be examined separately:

It should be emphasized that, although these simplified indices have been validated in individual studies, their reported values and proposed thresholds remain sensitive to acquisition parameters (vendor, field strength, pulse sequence, flip angle, timing of the hepatobiliary phase) and to post-processing methods. Consequently, reference values for normal and impaired liver function are not yet harmonized across MR platforms, and the cutoffs cited in the following subsections should be interpreted as center-specific reference points derived from the cohorts in which they were developed, rather than as universally validated clinical decision rules.

### Relative liver enhancement (RLE)

RLE is calculated from SI measurements obtained on unenhanced and HBP images using the following formula:

$$\:RLE\:\left(\%\right)=\:\left(\frac{SI\:liver\:HBP-SI\:liver\:unenhanced}{SI\:liver\:unenhanced}\right)\:\times\:100$$

In practice, RLE is obtained by placing regions of interest (ROIs) within the liver parenchyma, either at the segmental level or at the lobar level. ROIs typically measure 1–5 cm² and are carefully positioned to avoid vessels, focal lesions, and imaging artifacts [3, 13, 15, 18]. Mean RLE values are subsequently derived for analysis.

RLE has demonstrated a significant inverse correlation with MELD and MELD-Na scores, supporting its role as a quantitative imaging biomarker of hepatic function. Clinically, it has been investigated in scenarios such as predicting post-hepatectomy liver failure [15].

### Relative enhancement index (REI)

The REI is defined as the product of RLE in its unitless form (i.e., without multiplication of 100) and liver volume. As liver volume is an important variable to evaluate liver function, REI has been shown to demonstrate a stronger correlation with the plasma disappearance rate of ICG when compared with indices that do not incorporate volumetric data [19]. Beyond its association with established functional tests, REI has demonstrated clinical utility across multiple settings, including prognostic assessment in advanced CLD, evaluation of liver transplant candidates, prediction of post-transplant graft survival, and improved risk stratification for postoperative liver failure in patients undergoing hepatectomy [13].

$$\:REI=Vol\:liver\:\times\:\:\left(\frac{SI\:liver\:HBP-SI\:liver\:unenhanced}{SI\:liver\:unenhanced}\right)$$

### Hepatic uptake index (HUI)

The HUI reflects hepatocellular uptake of gadoxetate disodium on hepatobiliary phase (HBP) images while incorporating liver volume and correcting for the extracellular fluid (ECF) effect by using splenic SI. The ECF effect represents the distribution of contrast within the extracellular fluid space of abdominal organs after equilibrium is reached. This index has shown good correlation with ICG plasma disappearance rate [20]. ICG clearance test is a dynamic test of liver function in which an intravenously administered dye is selectively taken up by hepatocytes and excreted into the bile without undergoing metabolism, thereby reflecting hepatic uptake and excretory capacity [21]. The retention rate at 15 min (ICG-R15) corresponds to the fraction of dye remaining in the circulation after injection, with values greater than 20% indicating impaired hepatic function. In this context, an HUI threshold of 157.5 × 10⁻² identified patients with ICG-R15 > 20% among individuals with CLD or Child-Pugh class A cirrhosis, with 92% sensitivity and 94% specificity, a level commonly considered a contraindication to major hepatectomy [22]. The HUI is calculated as follows:

$$\:HUI=Vol\:liver\:\times\:\:\left[\left(\frac{SI\:liver\:HBP}{SI\:spleen\:HBP}\right)\:-1\right]$$

An additional metric derived from HUI, referred to as Relative Hepatic Uptake Index (rHUI), has been proposed for the assessment of hepatic function in the future liver remnant (FLR) prior to hepatectomy. In this approach, the volumetric component of the calculation is restricted to the portion of the liver that will remain after resection, thereby enabling a more targeted evaluation of the functional reserve of the remnant liver.

$$\:rHUI=Vol\:FLR\:\left[\left(\frac{SI\:FLR\:HBP}{SI\:spleen\:HBP}\right)\:-1\right]$$

### Signal intensity ratio (SIR)

The SIR is used to compare the SI of one tissue to another and helps to reduce variability related to scanner settings and acquisition techniques. For the assessment of liver function, SIR is calculated as the ratio between the SI of the liver and that of the paraspinal muscle on HBP images. Liver-to-muscle SIR has demonstrated a strong correlation with Child-Pugh and MELD scores and may help differentiate normal liver parenchyma from CLD [23].

$$\:SIR=\:\frac{SI\:liver\:HBP}{SI\:paraspinal\:muscle\:HBP}$$

### Contrast uptake index (CUI)

The contrast uptake index (CUI), also referred to in part of the literature as the contrast enhancement index (CEI), reflects liver function by comparing the SIR of the liver to a reference tissue (commonly paraspinal muscle) before and after contrast administration, using the following formula [3]:

$$\:CUI=\:\frac{SIR\:HBP}{SIR\:unenhanced}$$

### Liver-to-spleen index (LSI)

The liver-to-spleen index is calculated as the ratio of liver SI to splenic SI on post-contrast images. Because the spleen does not take up hepatocyte-specific contrast, it serves as a stable internal reference and allows LSI to reflect hepatocellular uptake efficiency.

$$\:LSI=\:\frac{SI\:liver\:HBP}{SI\:spleen\:HBP}$$

All these indices have demonstrated strong positive correlations with one another, along with good inter- and intraobserver agreement [24]. More detailed discussion of their clinical applications is provided in the dedicated Clinical Applications section.

A comparative overview of the quantitative indices and FLIS, summarizing their formulas, acquisition requirements, validated clinical applications, reported thresholds, and main limitations, is provided in Table 2.

**Table 2:** Comparative overview of quantitative indices and FLIS for liver function assessment on gadoxetic acid–enhanced MRI

Index	Formula	Reference tissue	Acquisition required	Validated clinical settings	Reported thresholds	Reproducibility	Major limitations
RLE *Relative Liver Enhancement*	(SI_liver HBP_ − SI_liver pre_)/SI_liver pre_ × 100	None (uses liver pre- and post-contrast SI)	Pre-contrast + 20-min HBP	Correlation with MELD/MELD-Na; prediction of post-hepatectomy liver failure (PHLF); graft function after transplantation	< 100 associated with PHLF risk [15]; inverse correlation with MELD/MELD-Na [18]	Good inter- and intra-reader agreement [24]; sensitive to ROI placement	Sensitive to vendor, field strength, sequence, and flip angle; thresholds derived in single-center cohorts; does not incorporate liver volume
REI *Relative Enhancement Index*	Vol_liver_ × [(SI_liver HBP_ − SI_liver pre_)/SI_liver pre_]	None	Pre-contrast + 20-min HBP + liver volumetry	Prognostic assessment in advanced CLD; transplant candidate evaluation; prediction of post-transplant graft survival; PHLF risk stratification	< 100 identifies poor liver function (AUC 0.94; sensitivity 100%, specificity 76%) [13]	Good inter- and intra-reader agreement [24]; depends on accuracy of volumetric segmentation	Requires volumetric segmentation (time-consuming if manual); same acquisition-related variability as RLE; single-center derivation of cutoff
HUI *Hepatic Uptake Index*	Vol_liver_ × [(SI_liver HBP_ / SI_spleen HBP_) − 1]	Spleen (corrects for extracellular fluid effect)	20-min HBP + liver volumetry	Identification of patients with ICG-R15 > 20% in CLD or Child-Pugh A cirrhosis; preoperative risk stratification before major hepatectomy	157.5 × 10^− 2^ for ICG-R15 > 20%, with 92% sensitivity and 94% specificity [22]	Strong correlation with ICG plasma disappearance rate [20]; good inter- and intra-reader agreement [24]	Not applicable in patients with splenectomy or altered splenic perfusion; requires volumetric data; cutoff derived in single-center cohorts
rHUI *Relative Hepatic Uptake Index*	Vol_FLR_ × [(SI_FLR HBP_ / SI_spleen HBP_) − 1]	Spleen	20-min HBP + segmental volumetry of the future liver remnant (FLR)	Regional functional assessment of the FLR before major hepatectomy, particularly in heterogeneous liver function (e.g., portal vein thrombosis, tumor invasion); prediction of severe PHLF	Significantly lower in severe PHLF vs. no/mild PHLF (417 ± 203 vs. 834 ± 325) [16]	Depends on accuracy of segmental segmentation; same observer-agreement profile as HUI	Requires accurate segment-level volumetry; same splenic dependence as HUI; cutoff not externally validated
SIR *Signal Intensity Ratio*	SI_liver HBP_ / SI_paraspinal muscle HBP_	Paraspinal muscle	20-min HBP only	Strong correlation with Child-Pugh and MELD functional assessment in patients with splenectomy	The study was conducted with a small sample size; therefore, larger studies are required to establish reliable cutoff values [23]	Reduces variability related to scanner settings and acquisition by using an internal ratio	Muscle SI may vary with patient habitus, edema, and ROI placement; less validated than splenic-referenced indices
CUI *Contrast Uptake Index (referred to as CEI in MASLD literature)*	SIR_HBP_ / SIR_pre−contrast_	Paraspinal muscle (typical)	Pre-contrast + 20-min HBP	Differentiation of MASH from simple steatosis; functional stratification across the MASLD spectrum	< 1.66 for MASH; >2.00 for isolated steatosis [39]; biopsy potentially avoidable in ~ 40% of patients [41]	Good correlation with histologic injury and inflammation [6, 40]	Acquisition- and post-processing–sensitive; dual-cutoff design leaves an indeterminate range; single-center derivation
LSI *Liver-to-Spleen Index*	SI_liver HBP_ / SI_spleen HBP_	Spleen	20-min HBP only (volume-LSR also requires liver and spleen volumetry)	Surrogate of hepatocellular uptake; correlation with CP, MELD, and laboratory function markers; volumetric variant (volume-LSR) for transplant and PHLF assessment	Volume-LSR shows high reproducibility and strong correlation with liver function scores [12]	Strong positive correlation with other indices [24]; good inter- and intra-reader agreement	Not reliable in splenectomy or altered splenic perfusion; SI ratios remain vendor- and sequence-dependent
FLIS *Functional Liver Imaging Score*	Sum of three HBP features (0–2 each): liver parenchymal SI vs. kidney; portal vein SI vs. liver; biliary contrast excretion. Range: 0 (worst) to 6 (best)	Right kidney (for parenchymal SI); portal vein (vs. liver)	20-min HBP only; purely visual assessment — no SI measurement or volumetry required	Prognosis in CLD (decompensation, ACLF, transplant-free survival, mortality); post-hepatectomy outcomes; post-transplant graft survival; post-TACE liver failure prediction; risk stratification in PSC	< 5 independently predicts decompensation in CP-A; ≥5 and ≥ 3 stratify functional stages [17]; <4 distinguishes preserved vs. impaired function and predicts transplant-free survival [10]; FLIS 6 associated with improved RFS/OS after hepatectomy [14]; FLIS 0 associated with 6.5% post-transplant survival [11]	Strong inter-reader agreement [10, 24]; qualitative nature makes it inherently less sensitive to acquisition variability	Less granular than continuous indices; subjective component in borderline cases; published cutoffs derived from a limited number of cohorts and not yet validated in large multicenter studies

ACLF, acute-on-chronic liver failure; CEI, contrast enhancement index; CLD, chronic liver disease; CP, Child-Pugh; FLR, future liver remnant; HBP, hepatobiliary phase; ICG, indocyanine green; MASH, metabolic dysfunction–associated steatohepatitis; MASLD, metabolic dysfunction–associated steatotic liver disease; MELD, Model for End-Stage Liver Disease; OS, overall survival; PHLF, post-hepatectomy liver failure; PSC, primary sclerosing cholangitis; RFS, recurrence-free survival; ROI, region of interest; SI, signal intensity; TACE, transarterial chemoembolization; volume-LSR, volumetric liver-to-spleen ratio

## Qualitative assessment: functional liver imaging score (FLIS)

**Fig. 1:**
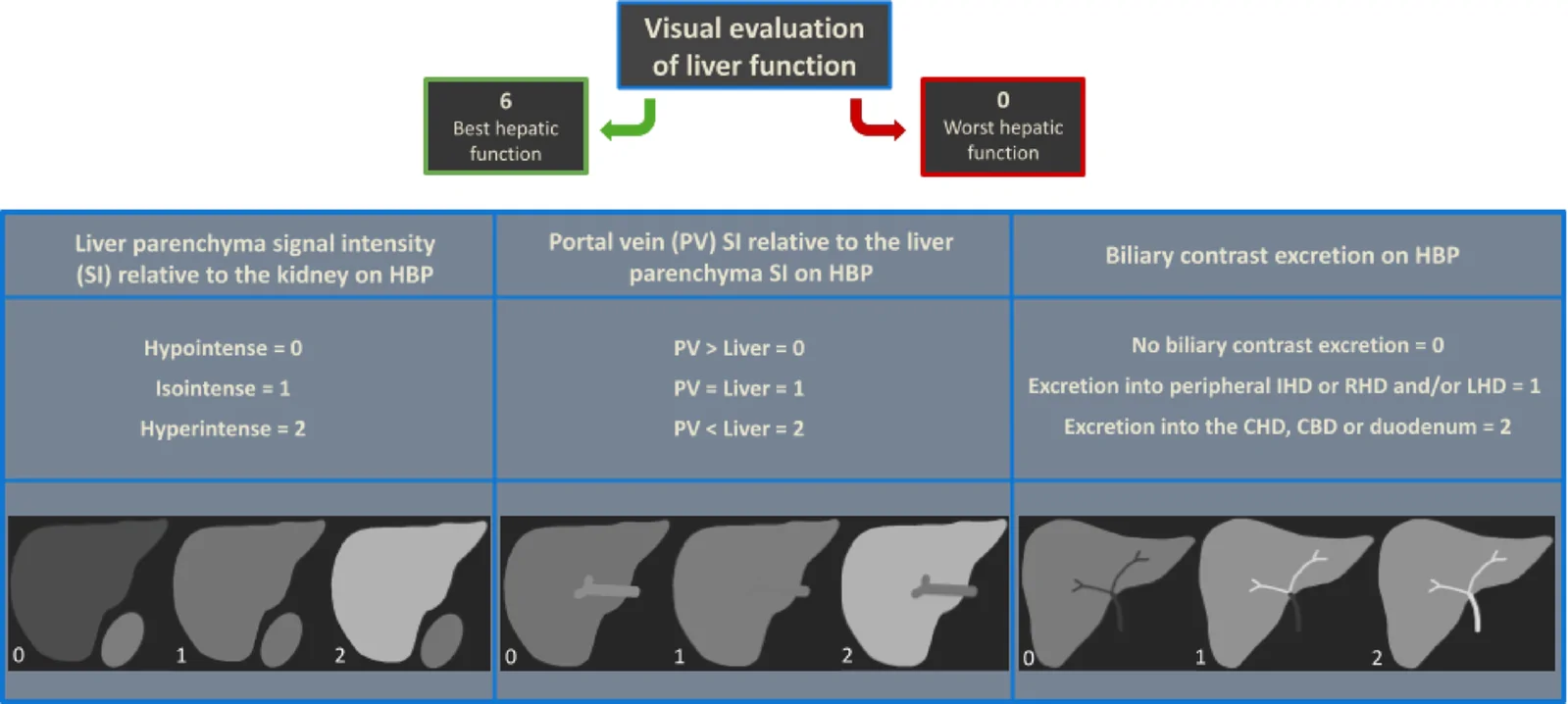
Schematic illustration of the FLIS

 Functional liver imaging score is based on a simple visual evaluation of liver parenchyma enhancement and biliary contrast excretion in the HBP, with no need to measure SI or to perform complex calculations [10]. The score scale ranges from 0 (worst) to 6 (best) and is based on three HBP features, each scored with 0, 1, or 2 points, as follows (Fig. 1):

1. Liver parenchyma SI relative to the right kidney on HBP, scoring 0, 1, or 2 points if the liver SI was hypo-, iso- or hyperintense to the kidney, respectively;
2. Portal vein (PV) SI relative to liver parenchyma SI on HBP, scoring 0, 1, or 2 points if the portal vein SI was hyper-, iso-, or hypointense to the liver, respectively;
3. Biliary tract contrast excretion assessment, scoring 0, 1, or 2 points if there is no excretion; excretion is seen in peripheral intrahepatic bile ducts, or the right and/or left hepatic duct(s); or the contrast excretion reaches the common hepatic duct, the common bile duct, or the duodenum, respectively.

FLIS is a reproducible qualitative score, easily incorporated into clinical practice, with strong inter reader agreement, and prognostic value in multiple CLD contexts. Although its qualitative nature makes FLIS inherently less sensitive to acquisition variability than signal intensity–derived indices, the cutoffs that have been proposed for clinical stratification (e.g., ≥ 5, ≥3, < 5, <4) derive from a limited number of cohorts [10, 17], and broader multicenter validation across vendors and field strengths is still needed before they can be universally adopted as decision rules.

## Clinical applications

### Prognosis and risk prediction in chronic liver disease

CLD is a major global cause of morbidity and mortality worldwide, progressing from fibrosis to cirrhosis and liver failure [25, 26]. Although liver biopsy remains the reference standard for fibrosis staging, its invasiveness, complications, and sampling variability limit its use, making noninvasive approaches, such as serum-based scores (e.g., fibrosis-4, enhanced liver fibrosis, metabolic dysfunction-associated steatotic liver disease related indices) and imaging techniques like transient elastography and magnetic resonance elastography, preferable for assessment [27]. MRI serves as a comprehensive “one-stop-shop” modality in CLD, enabling morphological evaluation, hepatocellular carcinoma (HCC) surveillance, fibrosis assessment, and functional analysis through GA-enhanced imaging, which is valuable in surgical and interventional planning, including pre- and post-hepatectomy, post-transplant evaluation, before locoregional therapies, such as ablation or transarterial chemoembolization (TACE).

REI, derived from GA-enhanced MRI, correlates strongly with established liver function metrics (CP, MELD, and ICG), with a cutoff < 100 accurately identifying poor liver function (AUC 0.94; 100% sensitivity, 76% specificity), supporting its use as a noninvasive functional biomarker [13].

FLIS is a simple and reproducible imaging marker with strong prognostic value in CLD. It independently predicts hepatic decompensation and mortality, with a cutoff of four points distinguishing preserved versus impaired liver function and predicting transplant-free survival [10]. Prognostic performance improves when FLIS is combined with splenic size, enhancing risk stratification for decompensation, acute-on-chronic liver failure, and mortality [9]. FLIS also correlates with CP class with cutoff ≥ 5 and ≥ 3 stratifying functional stages. FLIS < 5 independently predicts hepatic decompensation even in CP A patients, supporting its role as a simple and reliable imaging biomarker of liver function [17].

### Surgical planning and post-hepatectomy liver failure

Preoperative assessment of hepatic volume and function, traditionally based on clinical scores such as CP, is essential to minimize the risk of post-hepatectomy liver failure (PHLF), which results from an insufficient functional liver remnant. GA-enhanced MRI has emerged as a valuable tool for preoperative functional assessment, demonstrating strong correlations with postoperative outcomes [14, 15].

FLIS has been shown to provide prognostic information, with a score of 6 associated with improved recurrence-free and overall survival after hepatectomy for HCC, whereas lower scores (2–5) indicate poorer outcomes and warrant careful surgical consideration [14]. Quantitative imaging parameters further enhance risk stratification by integrating functional and volumetric assessment of the future liver remnant. For instance, reduced RLE (cut off < 100) on HBP imaging is associated with an increased risk of PHLF [15], and systematic reviews report moderate to high predictive accuracy of such metrics [28].

Importantly, GA-enhanced MRI enables regional functional assessment, which is particularly relevant in cases of heterogeneous liver function, such as portal vein thrombosis or tumor invasion. Parameters such as rHUI and rHUI/HUI have been identified as independent predictors of severe PHLF. The results demonstrated that patients who developed severe liver failure showed significantly lower rHUI values compared with those with no or mild PHLF (417 ± 203 vs. 834 ± 325) [16]. Additionally, the volumetric-based liver-to-spleen ratio (volume-LSR), derived from 3D volumetric analysis, has demonstrated high reproducibility and strong correlations with liver function scores, laboratory markers, and histological findings, supporting its role as a reliable imaging biomarker [12].

### Transplant evaluation

Liver transplantation is the definitive treatment for end-stage liver disease and selected hepatic malignancies, significantly improving long-term survival. However, recipients remain at risk for graft dysfunction, rejection, and vascular or biliary complications, requiring close monitoring. In this setting, GA-enhanced MRI provides a noninvasive means to assess graft function and predict outcomes [11, 15, 29].

Impaired hepatobiliary contrast excretion has been strongly associated with worse graft function, higher laboratory markers of liver injury, and increased MELD. This translates into significantly poorer outcomes, with markedly reduced 1-year retransplantation-free survival compared to patients with preserved excretion. Quantitative parameters such as RLE further refine prognostication, acting as independent predictors of graft failure and correlating with hepatocellular injury and survival [15].

The FLIS has also been validated as a robust qualitative predictor of outcomes after transplantation, outperforming most clinical and laboratory parameters. Higher FLIS values (4 to 6 points) are associated with excellent survival, whereas a FLIS of 0 identifies high-risk patients, with a survival probability of only 6.5% [11].

Overall, GA-enhanced MRI offers a reproducible and comprehensive tool for early detection of graft dysfunction and risk stratification, potentially guiding clinical decision-making and the need for further interventions such as biopsy.

### Locoregional therapies and interventional risk

When surgery is not feasible for hepatic tumors, particularly HCC and metastases, locoregional therapies, such as ablation and TACE are widely used, especially in patients with impaired liver function [30, 31]. However, these procedures carry risks, including vascular and biliary injury, as well as ischemic damage to non-tumoral liver parenchyma, which may lead to post-procedural liver failure [31–33]. In this context, GA-enhanced MRI plays a key role in preprocedural risk stratification by assessing baseline liver function. Pretreatment GA-enhanced MRI can also predict therapeutic response and outcomes. Heterogeneous HBP SI has been identified as an independent predictor of incomplete response to TACE, likely reflecting tumor heterogeneity and variable transporter expression linked to drug resistance, while elevated alpha-fetoprotein levels predict shorter disease-free survival [34].

Beyond risk prediction, GA-enhanced MRI is valuable for post-treatment evaluation, improving the differentiation between viable tumor and treatment-related changes. HBP hypointensity after ablation is associated with recurrence risk, and in TACE or transarterial radioembolization (TARE) it helps detect residual disease when arterial enhancement is inconclusive. DWI further complements assessment, with increased ADC values indicating treatment-induced necrosis [35].

More recently, FLIS-based models have been proposed to predict post-TACE liver failure, with FLIS, portal vein thrombosis, and INR identified as independent predictors. These models demonstrate superior diagnostic performance compared to conventional scores (ALBI, MELD and CP), supporting their role in clinical decision-making [32].

### Metabolic dysfunction associated steatotic liver disease

Metabolic dysfunction-associated steatotic liver disease (MASLD) has emerged as the leading cause of CLD worldwide, driven by the global rise in obesity and affecting approximately one-third of the adult population [36, 37]. A 2023 international consensus updated the nomenclature, replacing NAFLD and NASH with MASLD and metabolic dysfunction-associated steatohepatitis (MASH), respectively, and introducing broader categories such as steatotic liver disease (SLD), alcohol-associated liver disease (ALD), and MetALD, which is MASLD associated with increased alcohol intake [38, 39]. Several of the gadoxetic acid-enhanced MRI studies discussed in this section predate the 2023 consensus and therefore use the prior nomenclature (NAFLD and NASH). For consistency with the published literature, the original terms have been preserved when directly reporting the cutoffs and findings of those studies, with the understanding that NAFLD and NASH refer to the same clinical entities now designated as MASLD and MASH, respectively.

MASLD encompasses a spectrum ranging from isolated steatosis to MASH, fibrosis, and cirrhosis, with important prognostic and therapeutic implications [39, 40]. Although liver biopsy remains the reference standard for distinguishing these entities, its invasiveness and associated risks limit its widespread use [41]. Conventional imaging modalities (US, CT, MRI) can detect steatosis and complications but are limited in differentiating isolated steatosis from MASH [6, 40, 41].

GA-enhanced MRI has emerged as a promising noninvasive tool for functional assessment and disease stratification. Studies have shown that HBP enhancement reflects hepatocellular function and correlates with histologic injury rather than fat content alone. Quantitative parameters such as the contrast enhancement index (CEI), mathematically equivalent to the CUI described in Sect. “Contrast uptake index (CUI)”, are significantly lower in MASH compared to isolated steatosis [6, 40]. By establishing dual cut-off values (CEI < 1.66 for NASH and > 2.00 for isolated steatosis), Amorim et al. showed that invasive liver biopsies could potentially be avoided in 40% of the study population [41]. Similarly, reduced relative liver enhancement (RLE) and liver enhancement ratios have been associated with inflammation, hepatocellular ballooning, and fibrosis severity [6, 40].

Overall, GA-enhanced MRI-derived functional metrics provide a robust, noninvasive means to identify active liver injury and stratify disease severity across the MASLD spectrum.

### Primary sclerosing cholangitis and disease monitoring

Primary sclerosing cholangitis (PSC) is a rare, chronic, and progressive inflammatory disease of the intra- and extrahepatic bile ducts that leads to biliary injury, fibrosis, and cirrhosis. It is also associated with an increased risk of cholangiocarcinoma, requiring close surveillance. MRCP is the standard noninvasive imaging modality for evaluating biliary morphology in PSC [42–44].

Beyond structural assessment, GA-enhanced MRI enables functional evaluation of the liver parenchyma and may provide prognostic information. Studies have shown that HBP relative enhancement (RE) correlates strongly with established clinical scores (Mayo Risk Score, MELD, Amsterdam-Oxford Model) and laboratory markers of liver function. Higher RE values and preserved biliary excretion are associated with better transplant-free survival and lower disease severity. Conversely, reduced RE, particularly below a cutoff of 0.43, predicts impaired biliary excretion and worse outcomes, with progressive decline over time reflecting disease progression, especially in atrophic segments or those with severe ductal abnormalities [45].

Overall, GA-enhanced MRI offers a reproducible, noninvasive approach to assess regional liver function and heterogeneity, supporting risk stratification and clinical management in PSC.

## Case-based review (examples of reporting-style integration)

Examples demonstrating the use of volumetry combined with functional indices and FLIS are presented to highlight practical situations where GA-MRI offers advantages.

## Case 1: cholangiocarcinoma after portal vein embolization and hepatic vein deprivation

**Fig. 2:**
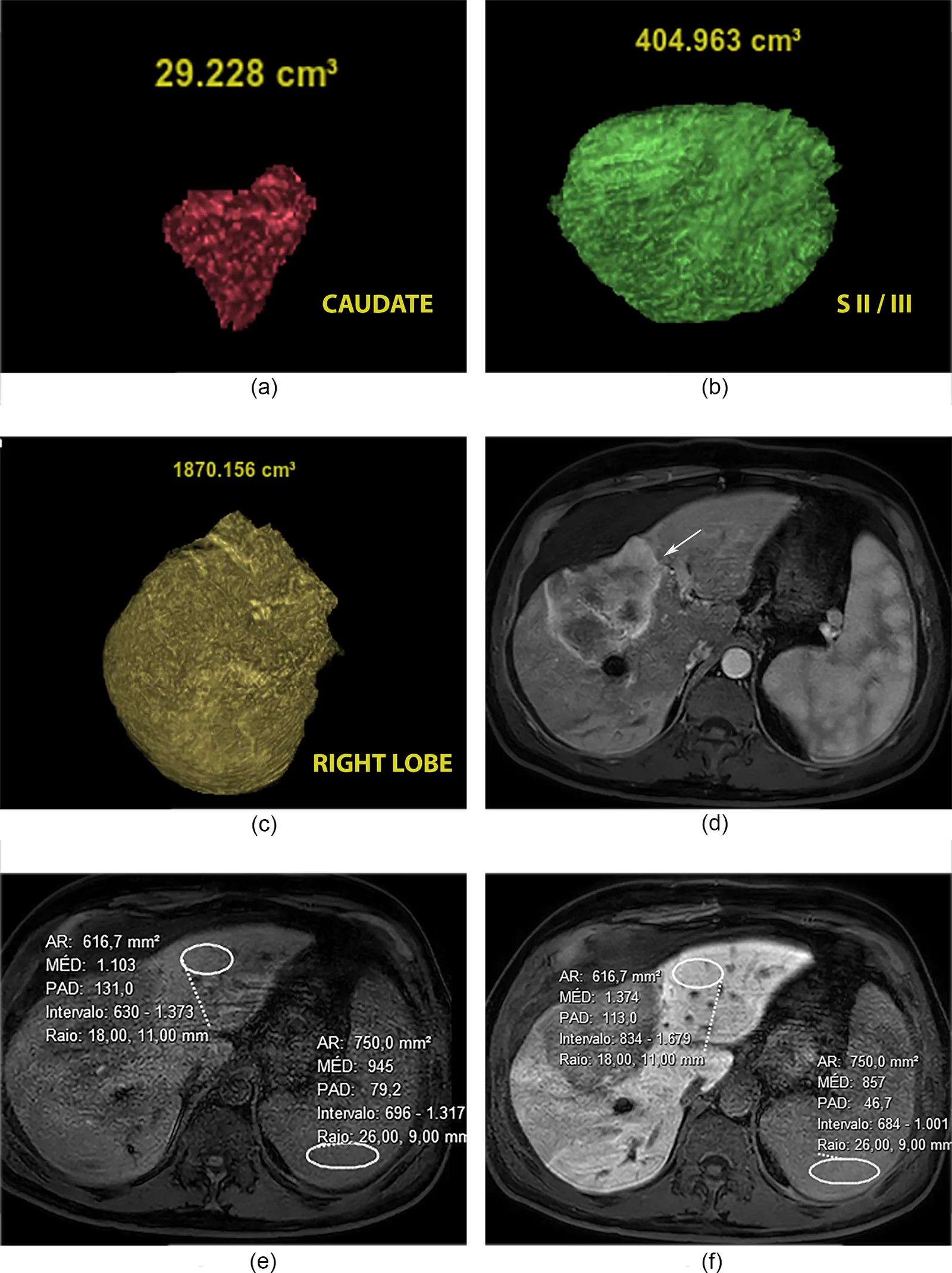
**a-f** GA-enhanced MRI with liver volumetry reconstruction.** A **Caudate lobe volume of 29 cm³. **B** Segments II and III volume of 405 cm³. **C** Segments V, VI, VIII, IVa and IVb volume of 1870 cm³. **D** T1-weighted axial arterial phase demonstrates cholangiocarcinoma (arrow) compromising segments IV, V and VIII. (**E**) Pre-contrast T1-weighted axial image showing liver remnant SI of 1103. **F** HBP after 20 min axial image showing liver remnant SI of 1374 and spleen SI of 857. The calculations were performed as follows: RLE = [(1374 - 1103)/1103] x 100 = 25; rHUI for segments I, II and III = 434 x [(1374 / 857) - 1] = 261

 A 66-year-old female patient with cholangiocarcinoma, who was a candidate for extended right hepatectomy underwent right hepatic vein deprivation and right portal vein embolization. GA-enhanced MRI was performed to evaluate the function of the future liver remnant (segments I, II and III). Quantitative parameters (RLE and rHUI) were calculated, along with the proposed remnant liver volume. The results indicated that extended right hepatectomy would not be safe due to the predicted risk of postoperative liver failure (Fig. 2).

## Cases 2–7: cirrhosis functional stratification with FLIS

These cases represent a cross-sectional clinical spectrum of chronic liver disease severity, ordered by advancing clinical dysfunction. Representative cirrhosis cases (MASH/hepatitis C virus (HCV)/alcohol-related) demonstrate stepwise decline in FLIS as MELD and CP worsens, including examples with absent biliary excretion, associated with low total FLIS.

### Case 2: **cirrhosis functional stratification with FLIS 6**.

**Fig. 3:**
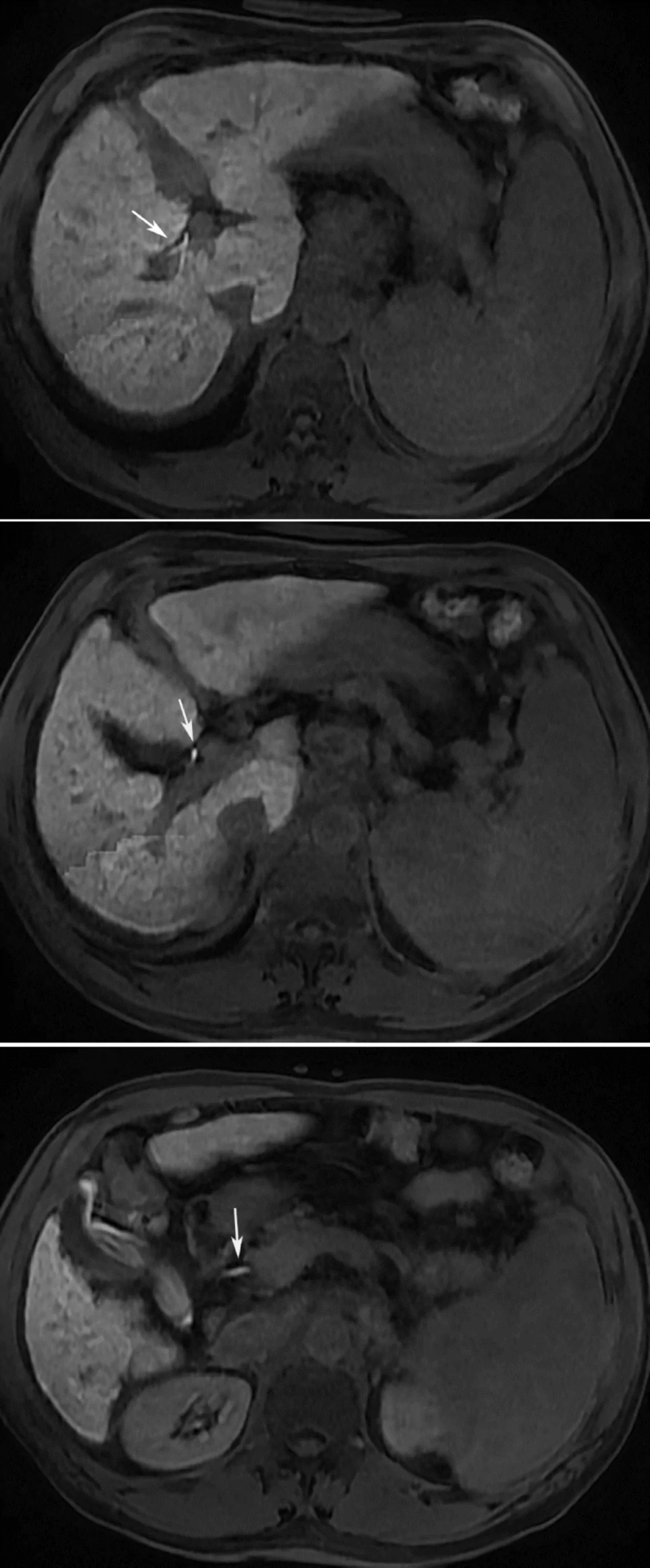
**a-c **HBP of GA-enhanced MRI.** A** Contrast excretion reaches extra-hepatic biliary ducts (arrows, a-c), scoring 2 points. **B** Liver SI is higher than that of the portal vein, scoring 2 points. **C** Liver SI is greater than that of the kidney, scoring 2 points. Total FLIS: 6 points

 Male, 60 years old, hepatic cirrhosis related to MASH, MELD 9, CP-B7 (Fig. 3).

### Case 3: **cirrhosis functional stratification with FLIS 5**.

**Fig. 4:**
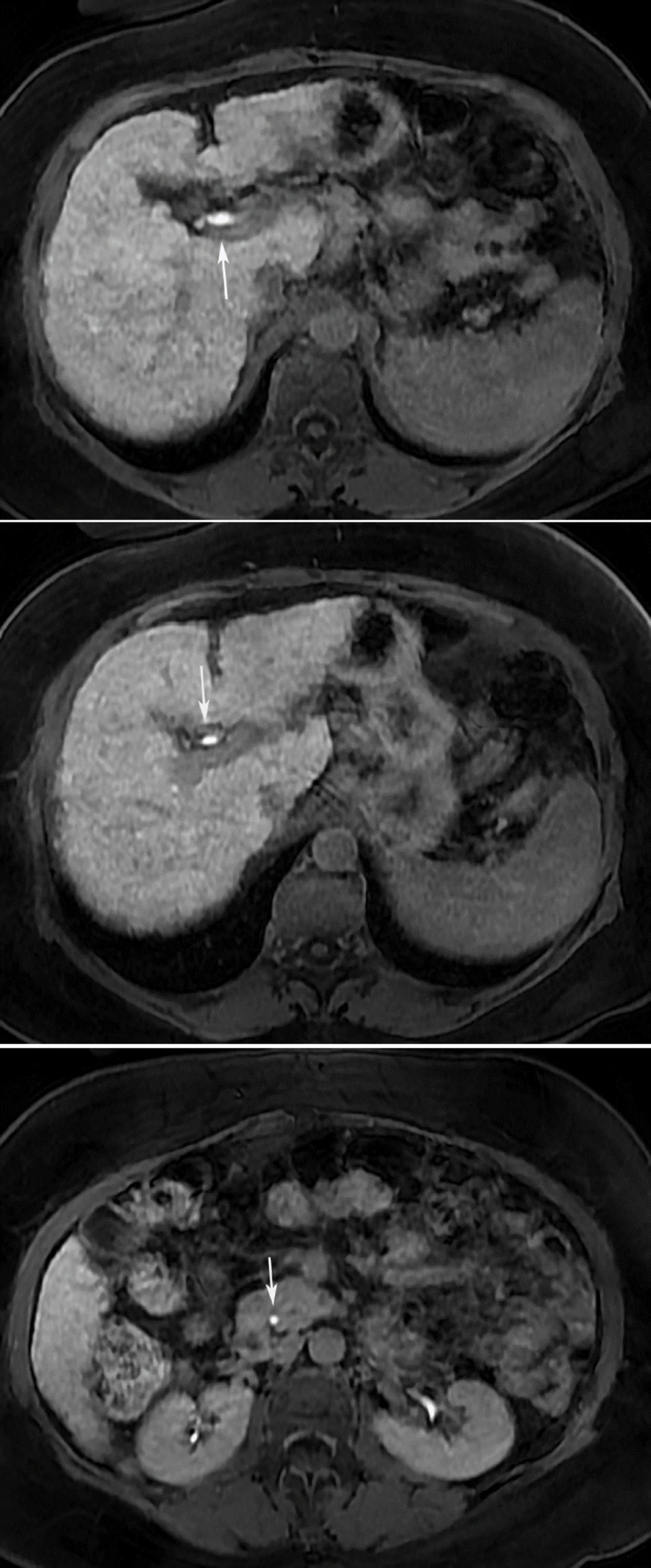
**a-c **HBP of GA-enhanced MRI.** A** Contrast excretion reaches extra-hepatic biliary ducts (arrows, a-c), scoring 2 points. **B** Liver SI is higher than that of the portal vein, scoring 2 points. **C** Liver SI is similar to that of the kidney, scoring 1 point. Total FLIS: 5 points

 Female, 56 years old, hepatic cirrhosis related to HCV, MELD 7, CP-A6 (Fig. 4).

### Case 4: **cirrhosis functional stratification with FLIS 4**.

**Fig. 5:**
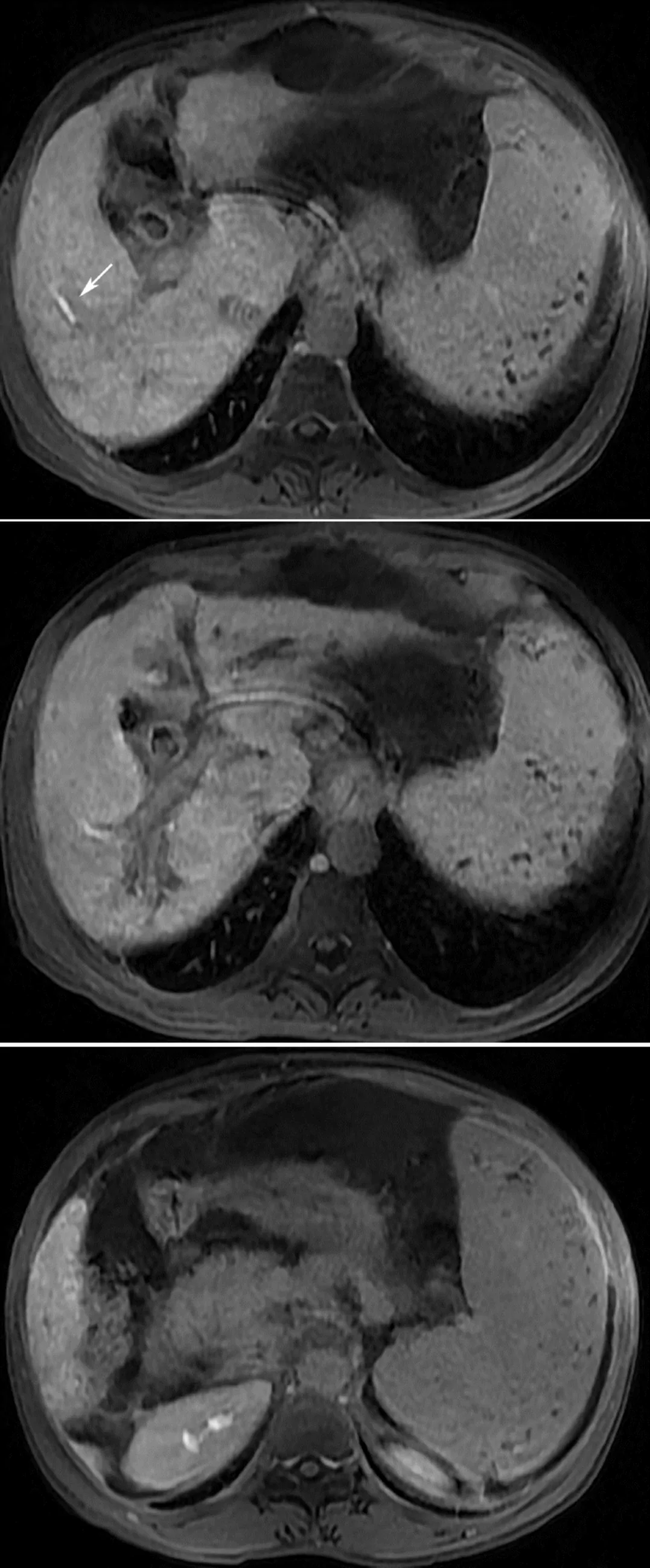
**a-c **HBP of GA-enhanced MRI.** A** Only peripheral intrahepatic contrast excretion is visualized (arrow), scoring 1 point. **B** Liver SI is higher than that of the portal vein, scoring 2 points. **C** Liver SI is similar to that of the kidney, scoring 1 point. Total FLIS: 4 points

 Female, 65 years old, hepatic cirrhosis related to HCV, MELD 17, CP-C10 (Fig. 5).

### Case 5: **cirrhosis functional stratification with FLIS 3**.

**Fig. 6:**
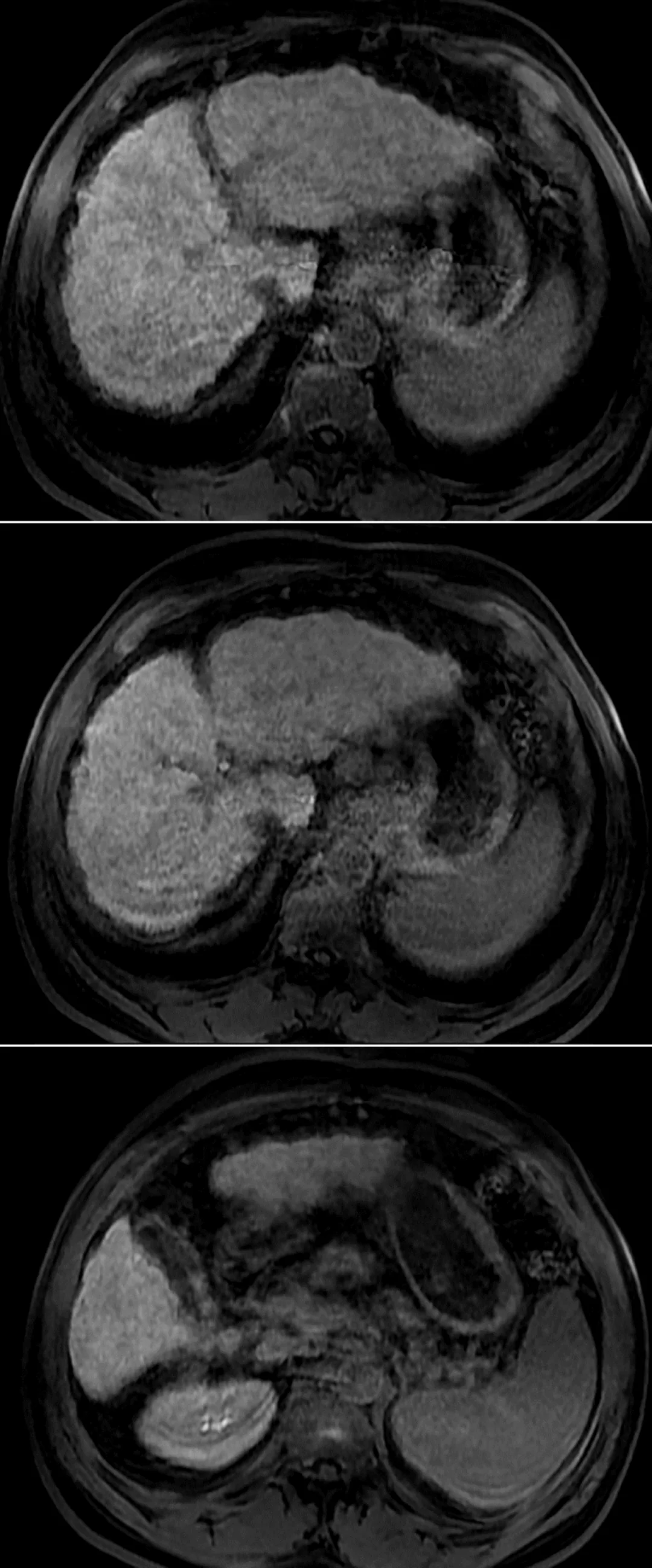
**a-c **HBP of GA-enhanced MRI.** A** No contrast excretion was visualized, scoring 0 points. **B** Liver SI is higher than that of the portal vein, scoring 2 points. **C** Liver SI is similar to that of the kidney, scoring 1 point. Total FLIS: 3 points

 Male, 52 years old, hepatic cirrhosis related to HCV, MELD 13, CP-C10 (Fig. 6).

### Case 6: **cirrhosis functional stratification with FLIS 2**.

**Fig. 7:**
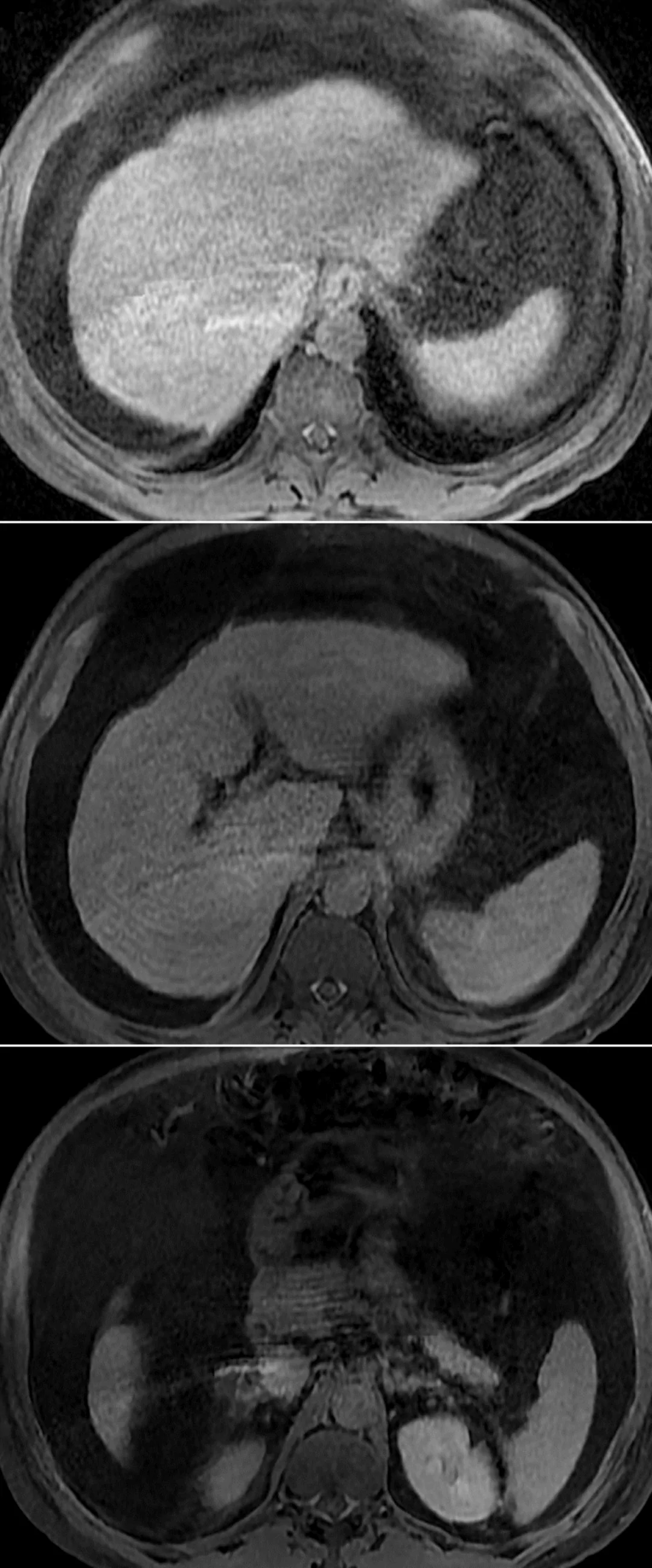
**a-c **HBP of GA-enhanced MRI.** A** No contrast excretion was visualized, scoring 0 points. **B** Liver SI is similar to that of the portal vein, scoring 1 point. **C** Liver SI is similar to that of the kidney, scoring 1 point. Total FLIS: 2 points

 Male, 60 years old, hepatic cirrhosis related to alcoholism, MELD 25, CP-B9 (Fig. 7).

### Case 7: **cirrhosis functional stratification with FLIS 1**.

**Fig. 8:**
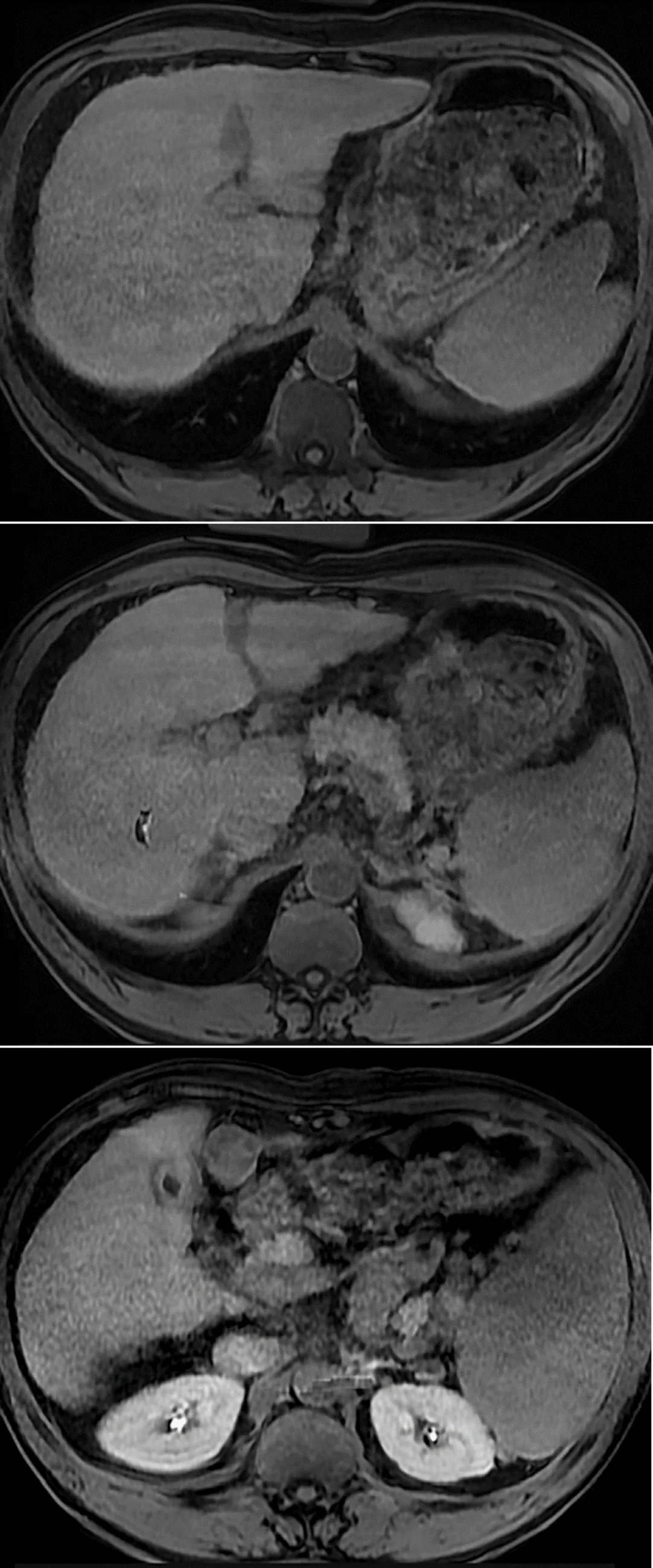
a-c HBP of GA-enhanced MRI.** A** No contrast excretion was visualized, scoring 0 points. **B** Liver SI is similar to that of the portal vein, scoring 1 point. **C** Liver SI is lower than that of the kidney, scoring 0 points. Total FLIS: 1 point

 Male, 47 years old, hepatic cirrhosis related to alcoholism, MELD 16, CP-B9 (Fig. 8).

## Future directions

Recent advances in GA-enhanced MRI are shifting beyond conventional quantitative parameters and FLIS toward AI-driven and multiparametric approaches for liver function assessment. The methods discussed below are promising but remain at an early stage of clinical translation, and the optimistic performance estimates reported in the literature must be read against the methodological constraints discussed throughout this section.

T1 mapping-derived metrics have shown strong correlations with established clinical scores such as ALBI, with post-contrast T1 values and hepatocyte uptake rates emerging as candidate noninvasive biomarkers of hepatic function [46]. However, absolute T1 values are known to depend on field strength, pulse sequence, inversion times, and B1 inhomogeneity, and reported reference ranges vary substantially across vendors and centers, which currently limits the use of single-threshold cutoffs in clinical practice. Expanding on this, whole-liver histogram and radiomics analyses capture intraparenchymal heterogeneity, by extracting quantitative features related to signal intensity distribution, texture, enhancement patterns, and spatial variation across the liver parenchyma. These features may reflect subtle functional impairment not fully appreciated by visual assessment or simple quantitative metrics, improving prediction of outcomes such as PHLF and outperforming traditional metrics. The reproducibility of radiomics features is nevertheless a well-recognized concern: many texture and higher-order features are sensitive to ROI delineation, voxel resampling, gray-level discretization, and reconstruction parameters, and only a minority remain stable across acquisition settings. [47]. Reported performance gains over traditional metrics therefore require independent confirmation in datasets acquired with different scanners and protocols before they can be translated into clinical use.

Deep learning has further enabled fully automated liver and spleen segmentation, facilitating rapid and reproducible extraction of volumetric and functional data. By reducing manual segmentation time and interobserver variability, AI-based tools may support standardized whole-liver assessment in both research and clinical workflows. In this context, the functional automated whole-liver score (FAWLS) represents a promising AI-derived biomarker, with reported superior performance compared with conventional measures (e.g., liver volume, ΔT1, FLIS) and strong correlation with clinical liver function scores. These results, however, are derived from single-center cohorts, and the generalizability of such models is constrained by training-set characteristics: differences in scanner manufacturer, field strength, sequence parameters, patient demographics, and disease etiology between the training set and a new clinical population can substantially degrade performance (domain shift). Independent external validation in geographically and technically diverse cohorts has not yet been reported for FAWLS, and, as with most deep-learning–based imaging biomarkers, limited explainability, that is, the difficulty of relating model outputs to interpretable physiological or imaging features, remains an obstacle to clinical adoption and regulatory clearance [29].

Additionally, integrated models combining imaging biomarkers with clinical variables have achieved high predictive performance, with AUC values up to 0.84, for post-hepatectomy liver failure, highlighting the value of multimodal, personalized risk stratification [48]. Such models may integrate radiomics features, quantitative MRI parameters, volumetric measurements, and laboratory data to provide a more comprehensive estimate of liver reserve and postoperative risk. It should nevertheless be emphasized that high-dimensional models combining many imaging and clinical variables in relatively small cohorts are at elevated risk of overfitting, that reported AUCs typically reflect internal cross-validation rather than prospective external testing, and that performance often drops meaningfully when such models are applied to independent populations. Reporting standards such as TRIPOD + AI and CLAIM, together with prospective multicenter validation, will be essential before these models can be considered for routine clinical use [49, 50].

Overall, these developments outline a plausible trajectory toward automated, whole-liver, radiomics-enhanced, and AI-based imaging biomarkers, enabling more comprehensive and individualized assessment of hepatic function in both surgical and transplant settings. Whether this trajectory translates into measurable clinical benefit will depend on the resolution of the methodological issues outlined above.

## Conclusion

GA-enhanced MRI has transformed liver imaging by extending evaluation beyond morphology to include noninvasive assessment of global and regional hepatic function. Quantitative parameters and the Functional Liver Imaging Score (FLIS) have shown the potential to improve risk stratification, treatment planning, and transplant evaluation in chronic liver disease.

More recently, research on T1 mapping, radiomics, and artificial intelligence has further explored functional assessment, with the potential to enable more reproducible, automated, and comprehensive evaluation across diverse clinical scenarios. These developments point toward multiparametric, patient-specific imaging biomarkers, but their integration into routine clinical decision-making remains contingent on the validation steps outlined in Sect. “Future directions”.

Despite this progress, key challenges persist, including the lack of standardization across vendors, field strengths, pulse sequences, and post-processing methods; substantial inter-platform variability in signal intensity-derived indices; the predominantly single-center origin of currently proposed cutoffs; and the need for large-scale prospective and multicenter external validation. Until these limitations are addressed, published thresholds should be regarded as center-specific reference points rather than universally applicable clinical decision rules. Addressing these limitations will be essential for the broader clinical adoption of these emerging biomarkers.

## Data Availability

No datasets were generated or analysed during the current study.

## References

[1] Lee, N.K., et al., *Biliary MR imaging with Gd-EOB-DTPA and its clinical applications*. Radiographics, 2009. 29(6): p. 1707–24.DOI: 10.1148/rg.296095501. 19959517

[2] Van Beers, B.E., C.M. Pastor, and H.K. Hussain, *Primovist, Eovist: what to expect?* J Hepatol, 2012. 57(2): p. 421–9.DOI: 10.1016/j.jhep.2012.01.031. 22504332

[3] Poetter-Lang, S., et al., *Quantification of liver function using gadoxetic acid-enhanced MRI*. Abdom Radiol (NY), 2020. 45(11): p. 3532–3544.DOI: 10.1007/s00261-020-02779-x. PMC7593310 33034671

[4] Cruite, I., et al., *Gadoxetate disodium-enhanced MRI of the liver: part 2, protocol optimization and lesion appearance in the cirrhotic liver*. AJR Am J Roentgenol, 2010. 195(1): p. 29–41.DOI: 10.2214/AJR.10.4538. 20566795

[5] Tsuda, N. and O. Matsui, *Cirrhotic rat liver: reference to transporter activity and morphologic changes in bile canaliculi–gadoxetic acid-enhanced MR imaging*. Radiology, 2010. 256(3): p. 767–73.DOI: 10.1148/radiol.10092065. 20663976

[6] Wu, Z., et al., *Usefulness of Gd-EOB-DTPA-enhanced MR imaging in the evaluation of simple steatosis and nonalcoholic steatohepatitis*. J Magn Reson Imaging, 2013. 37(5): p. 1137–43.DOI: 10.1002/jmri.23921. 23172731

[7] Guglielmo, F.F., D.G. Mitchell, and S. Gupta, *Gadolinium contrast agent selection and optimal use for body MR imaging*. Radiol Clin North Am, 2014. 52(4): p. 637–56.DOI: 10.1016/j.rcl.2014.02.004. 24889165

[8] Zhang, L., et al., *Detection of liver metastases on gadobenate dimeglumine-enhanced MRI: systematic review, meta-analysis, and similarities with gadoxetate-enhanced MRI*. Eur Radiol, 2019. 29(10): p. 5205–5216.DOI: 10.1007/s00330-019-06110-1. 30915560

[9] Bastati, N., et al., *Gadoxetic acid-enhanced MRI-derived functional liver imaging score (FLIS) and spleen diameter predict outcomes in ACLD*. J Hepatol, 2022. 77(4): p. 1005–1013.DOI: 10.1016/j.jhep.2022.04.032. 35525337

[10] Bastati, N., et al., *Does the Functional Liver Imaging Score Derived from Gadoxetic Acid-enhanced MRI Predict Outcomes in Chronic Liver Disease?* Radiology, 2020. 294(1): p. 98–107.DOI: 10.1148/radiol.2019190734. 31743083

[11] Bastati, N., et al., *Assessment of Orthotopic Liver Transplant Graft Survival on Gadoxetic Acid-Enhanced Magnetic Resonance Imaging Using Qualitative and Quantitative Parameters*. Invest Radiol, 2016. 51(11): p. 728–734.DOI: 10.1097/RLI.0000000000000286. 27175548

[12] Kudo, M., et al., *Evaluation of liver function using gadolinium-ethoxybenzyl-diethylenetriamine pentaacetic acid enhanced magnetic resonance imaging based on a three-dimensional volumetric analysis system*. Hepatol Int, 2018. 12(4): p. 368–376.DOI: 10.1007/s12072-018-9874-x. PMC6096956 29860678

[13] Eiras-Araujo, A.L., et al., *Relative enhancement index can be used to quantify liver function in cirrhotic patients that undergo gadoxetic acid-enhanced MRI*. Eur Radiol, 2023. 33(7): p. 5142–5149.DOI: 10.1007/s00330-023-09402-9. 36651953

[14] Sakai, N., et al., *Low functional liver imaging score is associated with poor prognosis following hepatectomy for hepatocellular carcinoma*. Sci Rep, 2024. 14(1): p. 31290.DOI: 10.1038/s41598-024-82741-9. PMC11682446 39732922

[15] Wibmer, A., et al., *Liver transplantation: impaired biliary excretion of gadoxate is associated with an inferior 1-year retransplantation-free survival*. Invest Radiol, 2012. 47(6): p. 353–8.DOI: 10.1097/RLI.0b013e318245f7be. 22543965

[16] Tsujita, Y., et al., *Prediction of post-hepatectomy liver failure using gadoxetic acid-enhanced magnetic resonance imaging for hepatocellular carcinoma with portal vein invasion*. Eur J Radiol, 2020. 130: p. 109189.DOI: 10.1016/j.ejrad.2020.109189. 32784077

[17] Lee, H.J., et al., *Validation of functional liver imaging scores (FLIS) derived from gadoxetic acid-enhanced MRI in patients with chronic liver disease and liver cirrhosis: the relationship between Child-Pugh score and FLIS*. Eur Radiol, 2021. 31(11): p. 8606–8614.DOI: 10.1007/s00330-021-07955-1. 33881570

[18] Ocal, O., et al., *Correlation of liver enhancement in gadoxetic acid-enhanced MRI with liver functions: a multicenter-multivendor analysis of hepatocellular carcinoma patients from SORAMIC trial*. Eur Radiol, 2022. 32(2): p. 1320–1329.DOI: 10.1007/s00330-021-08218-9. PMC8795026 34467453

[19] Yoneyama, T., et al., *Efficacy of liver parenchymal enhancement and liver volume to standard liver volume ratio on Gd-EOB-DTPA-enhanced MRI for estimation of liver function*. Eur Radiol, 2014. 24(4): p. 857–65.DOI: 10.1007/s00330-013-3086-5. 24356768

[20] Yamada, A., et al., *Quantitative evaluation of liver function with use of gadoxetate disodium-enhanced MR imaging*. Radiology, 2011. 260(3): p. 727–33.DOI: 10.1148/radiol.11100586. 21712472

[21] De Gasperi, A., E. Mazza, and M. Prosperi, *Indocyanine green kinetics to assess liver function: Ready for a clinical dynamic assessment in major liver surgery?* World J Hepatol, 2016. 8(7): p. 355–67.DOI: 10.4254/wjh.v8.i7.355. PMC4779164 26981173

[22] Yoon, J.H., et al., *Quantitative Assessment of Liver Function by Using Gadoxetic Acid-enhanced MRI: Hepatocyte Uptake Ratio*. Radiology, 2019. 290(1): p. 125–133.DOI: 10.1148/radiol.2018180753. 30375932

[23] Wang, Q., et al., *Quantitative evaluation of liver function with gadoxetic acid enhanced MRI: Comparison among signal intensity-, T1-relaxometry-, and dynamic-hepatocyte-specific-contrast-enhanced MRI- derived parameters*. Scandinavian Journal of Gastroenterology, 2022. 57(6): p. 705–712.DOI: 10.1080/00365521.2022.2032321. 35108168

[24] Beer, L., et al., *Inter- and intra-reader agreement for gadoxetic acid-enhanced MRI parameter readings in patients with chronic liver diseases*. Eur Radiol, 2019. 29(12): p. 6600–6610.DOI: 10.1007/s00330-019-06182-z. PMC6828941 31001679

[25] European Association for the Study of the, L., *EASL Clinical Practice Guidelines on non-invasive tests for evaluation of liver disease severity and prognosis – 2021 update*. J Hepatol, 2021. 75(3): p. 659–689.DOI: 10.1016/j.jhep.2021.05.025. 34166721

[26] Neshat SY et al (2021) Liver Disease: Induction, Progression, Immunological Mechanisms, and Therapeutic Interventions. Int J Mol Sci. 10.3390/ijms22136777 PMC8267746 34202537

[27] Singh S et al (2015) Diagnostic performance of magnetic resonance elastography in staging liver fibrosis: a systematic review and meta-analysis of individual participant data. Clin Gastroenterol Hepatol 13(3):440-451e6. 10.1016/j.cgh.2014.09.046 PMC4333001 25305349

[28] Wang, Q., et al., *Predictive value of gadoxetic acid-enhanced MRI for posthepatectomy liver failure: a systematic review*. Eur Radiol, 2022. 32(3): p. 1792–1803.DOI: 10.1007/s00330-021-08297-8. PMC8831250 34562137

[29] Chen, G., et al., *Quantitative assessment of liver graft function using functional automated whole-liver score derived from gadoxetic acid-enhanced MRI: a prospective cohort study*. Int J Surg, 2025. 111(8): p. 5235–5244.DOI: 10.1097/JS9.0000000000002566. 40576423

[30] Zhang, K., et al., *Gadoxetic Acid-enhanced MRI Scoring Model to Predict Pathologic Features of Hepatocellular Carcinoma*. Radiology, 2025. 314(2): p. e233229.DOI: 10.1148/radiol.233229. 39932410

[31] De Muzio F et al (2022) Complications after Thermal Ablation of Hepatocellular Carcinoma and Liver Metastases: Imaging Findings. Diagnostics (Basel). 10.3390/diagnostics12051151 PMC9139664 35626306

[32] Wu, X., et al., *Development and Validation of a Predictive Model for Liver Failure After Transarterial Chemoembolization Using Gadoxetic Acid-Enhanced MRI and Functional Liver Imaging Score*. Acad Radiol, 2025. 32(6): p. 3315–3323.DOI: 10.1016/j.acra.2024.12.068. 39809606

[33] European Association for the Study of the, L., *EASL Clinical Practice Guidelines on the management of hepatocellular carcinoma*. J Hepatol, 2025. 82(2): p. 315–374.DOI: 10.1016/j.jhep.2024.08.028. 39690085

[34] Lee, J.Y., et al., *Liver MRI and clinical findings to predict response after drug eluting bead transarterial chemoembolization in hepatocellular carcinoma*. Sci Rep, 2021. 11(1): p. 24076.DOI: 10.1038/s41598-021-01839-6. PMC8674226 34911966

[35] Gatti, M., et al., *Role of gadoxetic acid-enhanced liver magnetic resonance imaging in the evaluation of hepatocellular carcinoma after locoregional treatment*. World J Gastroenterol, 2022. 28(26): p. 3116–3131.DOI: 10.3748/wjg.v28.i26.3116. PMC9331537 36051340

[36] Tantu, M.T., et al., *Pathophysiology, noninvasive diagnostics and emerging personalized treatments for metabolic associated liver diseases*. npj Gut and Liver, 2025. 2(1): p. 18.DOI: 10.1038/s44355-025-00030-2.

[37] Chan, S.M., et al., *Imaging Evaluation for Steatotic Liver Disease*. Korean J Radiol, 2026. 27(2): p. 137–151.DOI: 10.3348/kjr.2025.0966. PMC12865115 41494787

[38] Osta, E.G., et al., *Current Update on Nomenclature, Diagnosis, and Management of Metabolic Dysfunction-associated Steatotic Liver Disease: Radiologists’ Perspective*. Radiographics, 2025. 45(12): p. e240221.DOI: 10.1148/rg.240221. 41231713

[39] Hosseini Shabanan, S., et al., *MASLD: What We Have Learned and Where We Need to Go—A Call to Action*. RadioGraphics, 2024. 44(11): p. e240048.DOI: 10.1148/rg.240048. PMC11580021 39418184

[40] Bastati, N., et al., *Noninvasive differentiation of simple steatosis and steatohepatitis by using gadoxetic acid-enhanced MR imaging in patients with nonalcoholic fatty liver disease: a proof-of-concept study*. Radiology, 2014. 271(3): p. 739–47.DOI: 10.1148/radiol.14131890. 24576046

[41] Amorim, V.B., et al., *Can gadoxetic acid-enhanced magnetic resonance imaging be used to avoid liver biopsy in patients with nonalcoholic fatty liver disease?* World J Hepatol, 2020. 12(9): p. 661–671.DOI: 10.4254/wjh.v12.i9.661. PMC7522564 33033571

[42] Steele, I.L., C. Levy, and K.D. Lindor, *Primary sclerosing cholangitis–approach to diagnosis*. MedGenMed, 2007. 9(2): p. 20. PMC1994832 17955076

[43] Morgan, M.A., et al., *Primary sclerosing cholangitis: review for radiologists*. Abdom Radiol (NY), 2023. 48(1): p. 136–150.DOI: 10.1007/s00261-022-03655-6. PMC9852001 36063181

[44] Khoshpouri, P., et al., *Imaging Features of Primary Sclerosing Cholangitis: From Diagnosis to Liver Transplant Follow-up*. Radiographics, 2019. 39(7): p. 1938–1964.DOI: 10.1148/rg.2019180213. 31626561

[45] Elkilany, A., et al., *Gadoxetic acid-enhanced MRI in primary sclerosing cholangitis: added value in assessing liver function and monitoring disease progression*. Abdom Radiol (NY), 2021. 46(3): p. 979–991.DOI: 10.1007/s00261-020-02731-z. PMC8257540 32918576

[46] Li, X., et al., *Evaluation of liver function using hepatocyte uptake and T1 mapping indices in gadoxetic acid-enhanced magnetic resonance imaging: correlation with the albumin-bilirubin grading system*. Quant Imaging Med Surg, 2025. 15(4): p. 3360–3371.DOI: 10.21037/qims-24-1827. PMC11994564 40235748

[47] Li, J., et al., *Predicting post-hepatectomy liver failure with T1 mapping-based whole-liver histogram analysis on gadoxetic acid-enhanced MRI: comparison with the indocyanine green clearance test and albumin-bilirubin scoring system*. Eur Radiol, 2025. 35(6): p. 3587–3598.DOI: 10.1007/s00330-024-11238-w. 39613961

[48] Jeong, B., et al., *Predicting post-hepatectomy liver failure in patients with hepatocellular carcinoma: nomograms based on deep learning analysis of gadoxetic acid-enhanced MRI*. Eur Radiol, 2025. 35(5): p. 2769–2782.DOI: 10.1007/s00330-024-11173-w. 39528755

[49] Mongan J, Moy L, Kahn CE Jr. (2020) Checklist for Artificial Intelligence in Medical Imaging (CLAIM): A Guide for Authors and Reviewers. Radiol Artif Intell 2(2):e200029. 10.1148/ryai.2020200029 PMC8017414 33937821

[50] Collins, G.S., et al., *TRIPOD + AI statement: updated guidance for reporting clinical prediction models that use regression or machine learning methods*. BMJ, 2024. 385: p. e078378.DOI: 10.1136/bmj-2023-078378. PMC11019967 38626948

